# A case of successful reperfusion through a combination of intracoronary thrombolysis and aspiration thrombectomy in ST-segment elevation myocardial infarction associated with an ectatic coronary artery

**DOI:** 10.1186/s12872-017-0527-0

**Published:** 2017-04-05

**Authors:** Yonggu Lee, Eunjin Kim, Bae Keun Kim, Jeong-Hun Shin

**Affiliations:** 1 Division of Cardiology, Department of Internal Medicine, Hanyang University Guri Hospital, 153, Gyeongchun-ro, Guri-si, Gyeonggi-do 11923 South Korea; 2 Department of Cardiology, Sungae Hospital, Seoul, Republic of Korea

**Keywords:** Myocardial infarction, Aspiration thrombectomy, Intracoronary thrombolysis, Coronary ectasia, Case report

## Abstract

**Background:**

Large thrombus burdens in ectatic coronary arteries that remain after aspiration thrombectomy can negatively impact outcomes following percutaneous coronary interventions in patients with acute myocardial infarction.

**Case presentation:**

A 53-year-old man presented with ST-segment elevation myocardial infarction (STEMI). Coronary angiography revealed an ectatic right coronary artery (RCA) that was completely occluded in the mid portion by a large amount of thrombus. Catheter-directed intracoronary thrombolysis with alteplase led to recovery of coronary blood flow, which multiple attempts of aspiration thrombectomy had failed to achieve. Coronary angiography 9 days later showed good blood flow and insignificant stenosis remaining in the RCA; this had completely resolved in 6 months’ follow-up coronary angiography.

**Conclusion:**

Catheter-directed intracoronary thrombolysis can be performed effectively and safely when repeat aspiration thrombectomy fails to produce satisfactory coronary reperfusion in STEMI patients with large thrombus burdens in ectatic coronary arteries.

## Background

Intracoronary thrombosis in patients with ST-segment elevation myocardial infarction (STEMI) can cause distal embolization, no-reflow phenomena and stent thrombosis, and increase the risk of adverse cardiac events and death following primary percutaneous coronary interventions (PCIs) [1, 2]. Although the beneficial effect of manual aspiration thrombectomy (MAT) during primary PCIs is still open to debate, it is frequently employed as a first-line therapy to reduce these adverse events [3]. However, there are no other effective options when MAT delivers insufficient coronary blood flow, especially in patients with large thrombus burdens. Here we report a case of successful coronary reperfusion through a combination of catheter-directed intracoronary thrombolysis and MAT in STEMI caused by thrombotic occlusion of an ectatic coronary artery.

## Case presentation

**Fig. 1:**
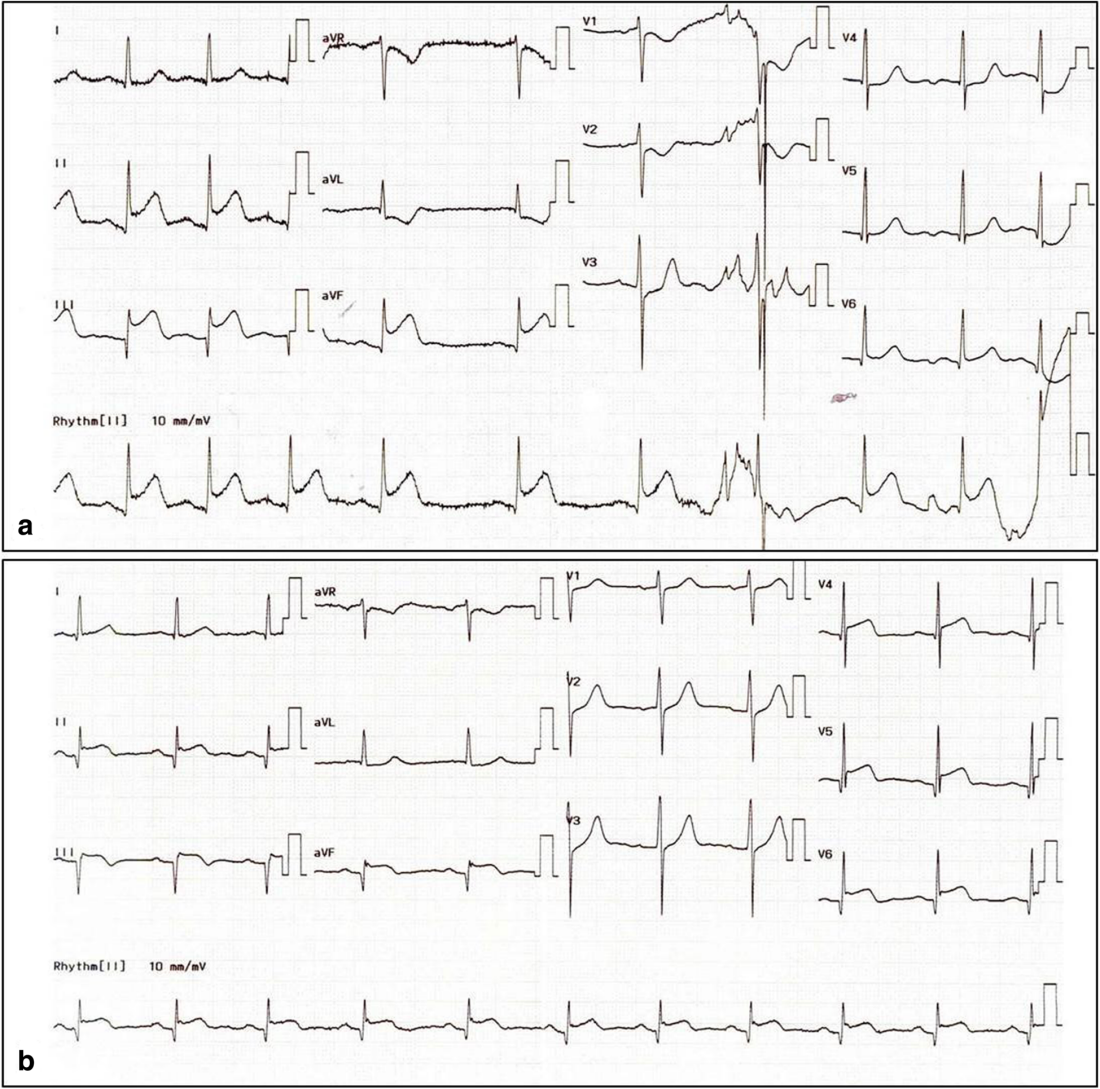
Electrocardiograms. **a** at admission. **b** after percutaneous coronary intervention

**Fig. 2:**
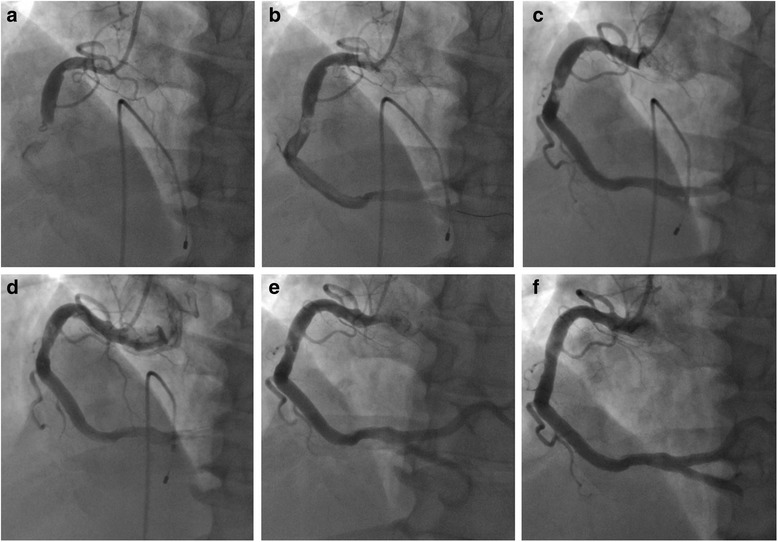
Coronary angiography. **a** Thrombotic total occlusion of the mid portion of the right coronary artery (RCA) with TIMI grade 0 flow. **b** After thrombus aspiration, a large filling defect remained due to extensive thrombus in the mid portion of the RCA with distal embolization in the posterior descending artery (PDA). **c** After initial intracoronary thrombolysis and repeated thrombus aspiration, improvement of TIMI flow, distal embolization, and residual thrombus at the mid portion of the RCA were noted. **d** After balloon angioplasty and second intracoronary thrombolysis, the culprit stenotic lesion was dilated, but TIMI flow worsened with distal embolization. **e** On the ninth day after the primary percutaneous intervention, TIMI flow was restored, but focal eccentric intermediate stenosis with some residual thrombus remained at the mid portion of the RCA. **f** Six months after discharge, marked dissolution of the thrombus and only minimal stenosis at the mid portion of the RCA was noted

**Fig. 3:**
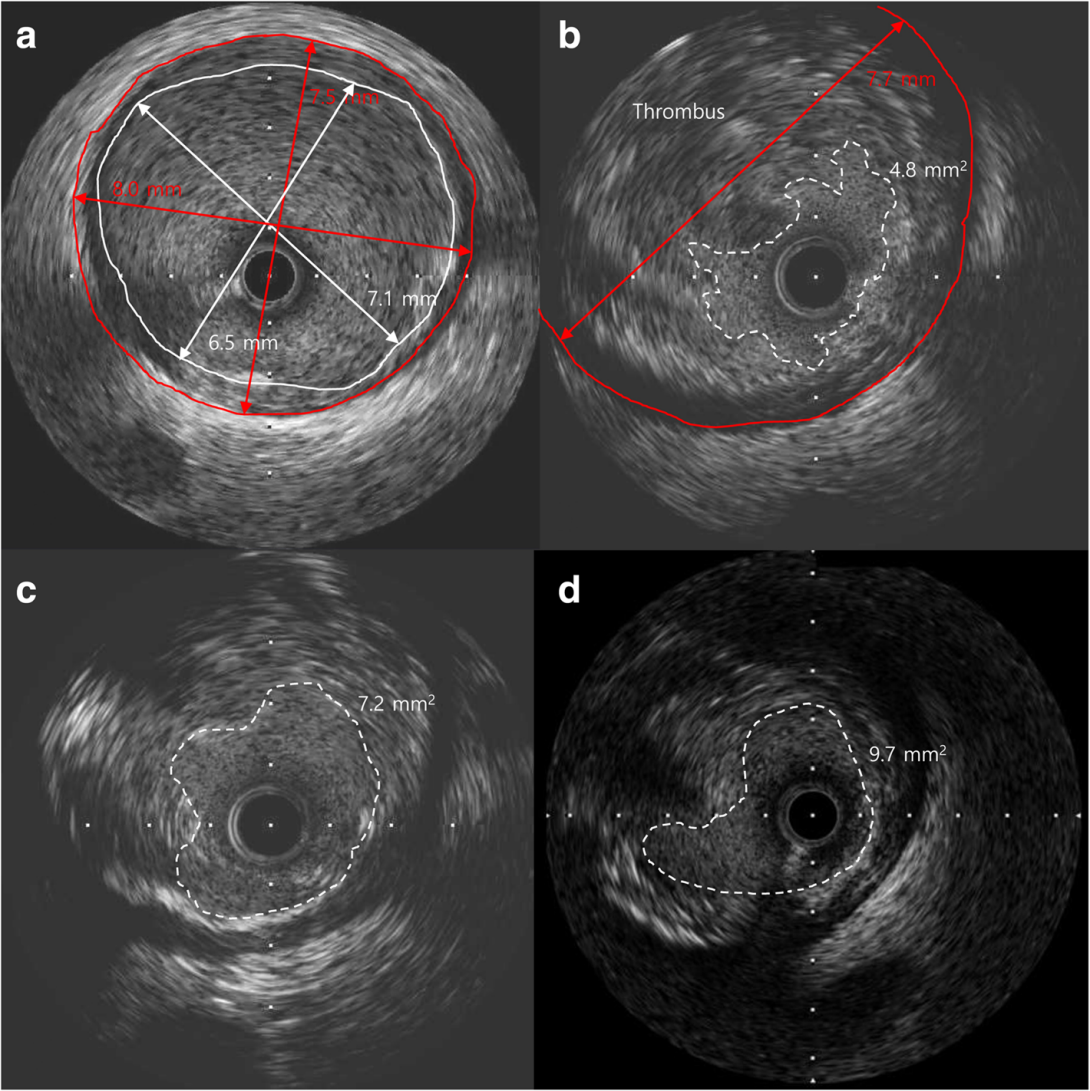
Intravascular ultrasound (IVUS) findings. **a** Adjacent normal segment proximal to the occlusion site. The shortest EEM and luminal diameter were 7.5 mm and 6.5 mm, respectively. **b** A ruptured plaque containing many necrotic components and a large amount of thrombus. The diameter of the external elastic membrane was 7.7 mm. **c** After thrombus aspiration and intracoronary thrombolysis, intermediate stenosis with remnant thrombi was noted. **d** On the ninth day after the primary percutaneous intervention, a minimal lumen area of about 9.73 mm^2^ with plaque was noted

A 53-year-old man presented in the emergency department with sudden chest pain lasting for 30 min. He was a 40-pack-year current smoker with high blood pressure on no medication. Blood pressure was 160/110 mmHg and pulse rate 60 beats/min. Electrocardiography showed ST-segment elevations in leads II, III, and aVF (Fig. 1a). Serum creatinine was 0.8 mg/dl and serum troponin I 0.01 ng/ml. Killip classification was class I. Aspirin 300 mg and ticagrelor 180 mg were administered, and coronary angiography (CAG) was performed immediately under temporary ventricular pacing. CAG revealed an ectatic right coronary artery (RCA) completely occluded by a large amount of thrombus in the mid-portion (Fig. 2a). A bolus of unfractionated heparin (8000 IU) and glycoprotein IIb/IIIa antagonist (abciximab, 0.25 mg/kg) was administered intravenously and MAT was performed three times using a 6-Fr aspiration catheter (Rebirth, Goodman Co. Ltd., Nagoya, Japan). After red thrombi were aspirated, thrombolysis in myocardial infarction (TIMI) grade 2 flow was achieved but a large filling defect persisted in the mid portion of the RCA, with distal embolization in the posterior descending artery (PDA) (Fig. 2b). Intravascular ultrasound (IVUS) (Atlantis, Boston Scientific, Natick, MA) revealed a ruptured plaque containing a large necrotic core and a large amount of thrombus remaining in the lesion. The external elastic membrane (EEM) diameter and the luminal diameter of the normal adjacent proximal segment of the occlusion were 7.5 mm and 6.5 mm, respectively (Fig. 3a). The culprit lesion was 7.7 mm in EEM diameter and 4.8 mm^2^ in minimal luminal area (MLA) (Fig. 3b). Because stent apposition might be difficult in such a large vessel, we decided to perform catheter-directed intracoronary thrombolysis using alteplase. The tip of a 2.7 Fr microcatheter (Progreat®, Terumo, Somerset, NJ, USA) was placed on the culprit lesion, and 5 mg of alteplase (Actilyse, Boehringer Ingelheim, Germany) in 5 mL normal saline was slowly administered over five minutes through the microcatheter. After 10 min, CAG showed improved coronary blood flow from the TIMI grade 2 to 3 in the mid portion of the RCA and from the TIMI grade 0 to 1 in the PDA, with remaining thrombi in the mid portion (Fig. 2c). Because significant stenosis persisted, a 4.5 × 8 mm non-compliant balloon (Quantum, Boston Scientific, Natick, MA) was inflated up to 16 atm in the mid portion of the RCA to disrupt the partially lysed thrombi. The lesion was dilated after the balloon angioplasty; however TIMI flow of the RCA appeared to be worsened (Fig. 2d). Intracoronary thrombolysis was repeated in the same manner. Blood flow improved to TIMI grade 3 and IVUS showed increased MLA with remaining thrombi (Fig. 3c). The chest pain was completely relieved and the ST-segment elevation was resolved (Fig. 1b).

Intravenous infusion of the glycoprotein IIb/IIIa inhibitor was maintained for 12 h after PCI. Oral administration of aspirin (100 mg/day), ticagrelor (180 mg/day), rosuvastatin (20 mg/day), bisoprolol (1.25 mg/day), and candesartan (8 mg/day) was continued. Low molecular weight heparin (dalteparin 100 IU/kg every 12 h; Fragmin®, Pfizer Inc., New York, NY) was administered subcutaneously after the femoral sheath was removed. No significant bleeding complications occurred after PCI. CAG repeated 9 days after the PCI revealed TIMI grade 3 blood flow in the RCA and PDA, and insignificant focal stenosis with a small amount of remaining thrombus in the lesion (Fig. 2e). IVUS showed that the MLA was 9.7 mm^2^ in the lesion (Fig. 3d) and the fractional flow reserve measured under maximal hyperemia was 0.98. Based on these assessments, we decided not to perform any additional intervention. The patient was discharged 12 days after PCI. CAG repeated 6 months after PCI showed complete dissolution of the thrombi and minimal remaining stenosis in the lesion with TIMI 3 blood flow in the RCA (Fig. 2f).

## Discussion and conclusions

Massive intracoronary thrombi are associated with unsuccessful angiographic reperfusion and unfavorable clinical outcomes [1, 4]. Unresolved intracoronary thrombi can cause microvascular obstruction, known as the no-reflow phenomenon, and result in reduced myocardial perfusion at the microvascular level, increased infarct size and higher mortality [5]. Although there have been improvements in antiplatelet and anticoagulant regimens and technical advances in PCIs, intracoronary thrombus remains one of the most dreaded enemies of interventional cardiologists. MAT is one of the most frequently used thrombectomy methods in primary PCIs, because the procedure is simple and the risk of vascular injury and distal embolism is low. Clinical guidelines also suggest that MAT is a reasonable approach when intracoronary thrombi are encountered [3]. However, studies have yielded inconsistent results in terms of its benefits in primary PCI [6–8]. The TASTE trial showed no benefits of MAT for mortality, re-hospitalization and stent thrombosis [8]. More recently, Jolly et al. [7] also reported that MAT did not reduce cardiovascular events, whereas it increased stroke rate. This result may partly be related to insufficient thrombus removal and inadequate coronary blood flow recovery in cases with massive intracoronary thrombosis. Safe and feasible alternative strategies are needed when MAT fails during primary PCI.

Before coronary stents were much used, intracoronary thrombolysis was used in patients with all types of coronary artery disease [9, 10]. However, because studies gave discouraging results [11] and primary PCI with stent implantation became routine, intracoronary thrombolysis was rarely used in clinical practice. In recent years, intracoronary thrombolysis has regained popularity as an adjuvant therapy for primary PCI, as studies using different thrombolytic agents and improved antiplatelet regimens showed it to be safe and effective. Kelly et al. [12] reported that intracoronary infusion of tenecteplase was safe and effective for coronary flow recovery in patients with myocardial infarction. More recently, Boscarelli et al. [13] found that adjuvant intracoronary infusion of low dose tenecteplase and alteplase in STEMI significantly reduced the thrombi remaining after MAT and improved coronary blood flow. Several case reports of massive intracoronary thrombosis also described successful recovery of coronary blood flow after intracoronary thrombolysis using alteplase [14] and tenecteplase [15].

We used the glycoprotein IIb/IIIa inhibitor after intracoronary thrombolysis, which may significantly increase the risk of major bleeding events. However, the thrombolytic agent doses used through intracoronary routes are usually much lower than those used through intravenous routes. Kelly et al. [12] also reported that only 1 case of major bleeding (2.9%) among 34 patients after intracoronary thrombolysis using tenectaplase, which is similar to the major bleeding rates reported regularly in acute coronary syndrome [16]. Moreover, the majority of the patients (76%) in their study received glycoprotein IIb/IIIa inhibitors simultaneously with intracoronary thrombolysis.

In our case, a massive thrombotic occlusion occurred in the ectatic RCA. Coronary ectasia is defined as a diffuse dilation of a coronary artery to a diameter at least 1.5 times larger than normal coronary artery diameter [17]. It is present in 1–5% of patients undergoing CAG [18]. Various reperfusion strategies including MAT alone, simple balloon angioplasty, pulse-spray thrombolysis, intracoronary thrombolysis and mesh-covered stent implantation have been proposed in STEMI in ectatic coronary arteries [19–21]. Several randomized controlled trials have reported that rheolytic thrombectomy was more effective than MAT in thrombus removal and myocardial reperfusion in patients with STEMI, although there were no differences in infarct sizes and adverse cardiac events following PCI between rheolytic thrombolysis and MAT [22, 23]. Simple balloon angioplasty might increase the risk of distal embolization after intracoronary thrombus is incompletely removed. Prolonged intravenous heparin infusion is a viable option for the remaining thrombus after MAT in ectatic coronary arteries [24]. However, for ectatic coronary arteries, because the sheer amount of thrombus is massive and blood flow is slow, no single strategy would be sufficient. In fact, we employed multiple strategies namely MAT, balloon angioplasty and intracoronary thrombolysis during the PCI to achieve a good immediate result. We think that the additional anatomical and physiological information obtained by IVUS and by measuring the fractional flow reserve helped us avoid stent implantation, which could have led to incomplete stent apposition. Self-expendable stents have been introduced for patients with complex coronary anatomy including aneurysmal dilation, which may also be an alternative strategy to avoid difficulty in stent apposition, as shown in our case [25].

In conclusion, catheter-directed intracoronary thrombolysis may be a safe and effective alternative reperfusion strategy that may be selected when MAT alone fails to achieve sufficient coronary blood flow in the culprit vessel in STEMI associated with massive thrombosis in ectatic coronary arteries.

## Abbreviations

CAG: Coronary angiography
IVUS: Intravascular ultrasound
MLA: Minimal lumen area
PCI: Primary percutaneous coronary intervention
PDA: Posterior descending artery
RCA: Right coronary artery
STEMI: ST-elevation myocardial infarction
TIMI: Thrombolysis in myocardial infarction
